# Editorial: Ventricular mechanics in congenital heart disease and pediatric cardiology

**DOI:** 10.3389/fped.2024.1433819

**Published:** 2024-06-04

**Authors:** Paolo Ciliberti, Marcello Chinali, Claudio Capelli

**Affiliations:** ^1^ Cardiology Unit, Bambino Gesù Children’s Hospital IRCSS, Rome, Italy; ^2^ Institute of Cardiovascular Science, UCL, London, United Kingdom

**Keywords:** echocardiography, CMR, congenital heart disease, ventricular mechanics, pediatric cardiology

**Editorial on the Research Topic** Ventricular mechanics in congenital heart disease and pediatric cardiology

In the dynamic realm of pediatric cardiology, the intricate mechanics of the ventricles stand as a focal point for both research and clinical practice. As editors of the Research Topic “Ventricular Mechanics in Congenital Heart Disease and Pediatric Cardiology”, we are privileged to present a collection of articles that primarily delve deep into this crucial aspect of cardiac physiology, offering insights that promise to enhance our understanding but also explore other multiple facets of caring for young patients with congenital heart conditions.

The ventricular system, with its complex interplay of myocardial fibers, valves, and chambers, orchestrates the intricate dance of blood flow essential for sustaining life. In the context of congenital heart disease (CHD), where structural abnormalities alter this delicate equilibrium, comprehending ventricular mechanics becomes paramount. From the simplest lesions like atrial or ventricular septal defect to the more complex ones such as single ventricle diseases like hypoplastic left heart syndrome, each condition presents unique challenges that require tailored approaches informed by rigorous research.

Our collection aimed to build upon the research selection “Ventricular Mechanics in Congenital Heart Disease”, previously published in this journal and edited by Giovanni Biglino and Adelaide de Vecchi (1). Their work, to which we contributed as authors, extensively explored the topic, analyzing its various facets and different diagnostic methodologies, including their most recent developments and potential innovations (2–4). Therefore, our goal with this new collection was not merely to provide a new comprehensive systematic treatment of the subject matter, but rather to complement it by highlighting specific cases and delving deeper into certain aspects within the different peculiar contexts.

Within this Research Topic, several articles shed light on various facets of ventricular mechanics in pediatric cardiology. Sjöberg et al. investigate how the left ventricle (LV) respond to intervention measured by non-invasive pressure-volume loops in patients with right ventricle (RV) volume load due to atrial septal defect or repaired Tetralogy of Fallot. Avesani et al. offer an elegant review that systematizes the role of echocardiography, including advanced techniques such as myocardial deformation imaging and 3D echo, in evaluating cardiac mechanics in the most significant CHDs. This systematization helps us organize the plethora of information present in the literature and understand how patients' unique anatomies, as different CHDs exhibit distinct anatomical characteristics, affect cardiac mechanics.

Iacobelli et al. provide a review aiming to delineate a systematic echocardiographic approach for pediatric subjects candidated for and supported by pulsatile-flow LV assist devices. Echocardiography plays a crucial role in determining eligibility for left ventricular assist device (LVAD) placement in patients experiencing advanced heart failure (HF) and in monitoring patient care after the implantation procedure. Due to its unique nature, the pediatric population and pulsatile-flow LVADs used in pediatrics require specific skills. Therefore, pediatric echocardiographers and the authors help us develop a systematic approach to image patients pre and post LVAD implantation.

Panebianco et al. present an unusual combination: Epstein-Barr virus and constrictive pericarditis, causing significant heart failure in a young man. Constrictive pericarditis is a rare chronic inflammatory process that, through a complex process of ventricular interdependence, can lead to heart failure if not diagnosed and treated correctly. Although Epstein-Barr virus related pericarditis is a very rare condition, it is important to recognize and consider the possibility of it, as it should still be included in the list of differential diagnoses.

Among the articles exploring other aspects, not strictly ventricular, of congenital heart diseases, Petreschi et al. report a large single-center experience in managing airway compression in patients with aortic arch malformations. The authors describe clinical presentation, instrumental findings, treatment, and follow-up of a large cohort of children affected by either complete vascular rings or anomalous origin of the Innominate artery, and they provide a useful and practical diagnostic and therapeutic algorithm.

Soliman et al. introduce a new entity of cardiovascular neurocristopathies. Cardiac neural crest (CNC) cells, originating from the dorsal neural tube, are pivotal architects of the cardio-neuro-vascular domain, which orchestrates the embryogenesis of critical cardiac and vascular structures. Diseases such as bicuspid aortic valve and thoracic aortic aneurysm share this common embryonic origin, and identifying the underlying pathogenic and genetic factors may be crucial for the care of these patients.

The studies unveil the intricate tapestry of congenital heart diseases, revealing their multifaceted nature and the depth of complexity within this domain, as evidenced by the unique settings that are presented and treated, and by the cases that are proposed.

From this overview of works, it becomes evident that although echocardiography remains the most widely used and readily available method, a comprehensive study of these patients, and in particular of their complex ventricular mechanics, must acknowledge the importance of incorporating advanced imaging techniques into daily practice. These include cardiac magnetic resonance imaging, CT scans, 3D modelling and reconstruction imaging, and advanced echocardiography techniques such as strain and speckle tracking (5–7).

As editors, we extend our appreciation to the authors for their valuable contributions to this Research Topic. These articles not only deepen our understanding of ventricular mechanics but also provide practical insights that can directly impact the care of young patients with congenital heart conditions.
